# Biochar Ingestion Is Associated with Hindgut Symbiotic Disruption and Mortality in *Coptotermes formosanus*: A Proposed Protist-to-Bacterial Cascade

**DOI:** 10.3390/insects17101044

**Published:** 2026-10-12

**Authors:** Jianan Zhao, Xinyi Xu, Jingyi Li, Jieting Ye, Tong Tong, Wenjing Wu, Zhibing Wan, Yong Chen

**Affiliations:** 1 College of Life and Environment Science, Huangshan University, Huangshan 245021, China; 2 Guangdong Key Laboratory of Animal Conservation and Resource Utilization, Guangdong Public Laboratory of Wild Animal Conservation and Utilization, Institute of Zoology, Guangdong Academy of Sciences, Guangzhou 510260, China

**Keywords:** biochar, *Coptotermes formosanus*, protist-bacterial symbiosis, gut microbiome, bait technology

## Abstract

Background: Biochar is being explored for pest management, but its potential as a subterranean termite bait and its mechanism of action on the gut symbiont community are poorly understood. Methods: We fed *Coptotermes formosanus* workers a diet of pine wood powder containing 0%, 5%, or 20% (*w*/*w*) rice-straw biochar. We then measured feeding preference, body weight, mortality, and analyzed changes in the hindgut flagellate community, bacterial 16S rRNA gene profiles, and host transcriptomics. Results: The 5% biochar diet did not deter feeding, whereas 20% reduced intake by ~18.75%. Both doses were associated with significant, time- and dose-dependent reductions in body weight and survival. The three major cellulolytic flagellate species (*Holomastigotoides mirabile*, *Pseudotrichonympha grassii*, *Spirotrichonympha leidyi*) declined sharply, coinciding with a restructuring of the bacterial community, notably a decrease in nitrogen-fixing Dysgonomonadaceae and an increase in opportunistic taxa. Host transcriptomics revealed exploratory, dose-dependent trends from anabolic growth toward catabolic stress and detoxification pathways, including upregulation of the proteasome and downregulation of ribosomal and energy metabolism pathways; several of these enrichment signals did not remain significant after multiple-testing correction and are therefore treated as hypothesis-generating. Conclusions: Our data are consistent with a proposed cascade hypothesis in which biochar ingestion is associated with disruption of essential gut flagellates, subsequent restructuring of bacterial symbiosis, and host metabolic stress. However, these links are inferred from parallel measurements and require continuous causal and rescue experiments for confirmation. Reduced intake at 20% and unmeasured biochar physicochemical properties remain alternative or non-exclusive explanations. Low-dose, non-deterrent biochar formulations represent candidate multi-mode active ingredients for termite bait development.

## Simple Summary

Termites cause billions of dollars in damage to wooden buildings each year. Current control methods often involve poisons that need to be shared among nestmates to work. This study tested whether a charcoal-like material called biochar, made from burnt rice straw, could be a safer alternative. We fed termites pine wood powder containing 0%, 5%, or 20% biochar. The 5% diet was not deterrent in a choice test, whereas 20% reduced intake. Over 30 days, both biochar diets were associated with reduced body weight and survival. Our data suggest that biochar ingestion is associated with a sequence of hindgut symbiont disruption: major cellulolytic protozoa declined, bacterial community structure changed, and host transcriptome showed exploratory stress/catabolic trends. However, because these responses were measured in parallel and no continuous causal or rescue experiment was performed, we cannot establish a definitive cascade. Reduced intake at the high dose and unmeasured biochar physicochemical properties remain alternative or contributing explanations. Because 5% biochar acted slowly and was not deterrent, it may be a promising candidate ingredient for termite baits, but mechanism and field tests are needed.

## 1. Introduction

Biochar is the product of biomass pyrolysis under limited oxygen conditions, currently employed as a soil amendment to sequester carbon, enhance soil fertility, prime plant defense systems, and improve crop yields [1,2,3,4,5,6,7]. Biochar also plays a certain role in herbivorous pest control. For example, the application of >5% biochar could decrease the fecundity of planthoppers on rice (*Oryzae sativa*), and >3% could decrease the fecundity of the English grain aphid (*Sitobion avenae*) on wheat (*Triticum* sp.) [2,8]. Additional studies have found that biochar can directly exert certain negative effects on soil organisms [9,10]. Although the exact mechanism by which biochar induces mortality in soil fauna remains unclear, some studies suggest that the ingestion of biochar particles by soil animals during feeding may be one possible cause of their death [10,11,12];. This finding implies that biochar could potentially be developed as a control agent for certain soil pests, thereby reducing the reliance on chemical pesticides.

The Formosan subterranean termite (*Coptotermes formosanus*) is a soil/wood-nesting termite and is highly destructive termite in China, Japan, and the USA. According to our earlier research, adding biochar to soil containing more than 10% (*w*/*w*) of biochar was able to significantly lower the fitness of *C. formosanus* [11]. However, we cannot rule out whether the observed reduction in fitness is due to alterations in the soil environment or to the ingestion of biochar particles. Considering the potential of biochar to be developed as a control agent for subterranean pests, we therefore used *C. formosanus* as our research subject. By incorporating biochar with pine wood powder as a phagostimulant and feeding this mixture to termites, we investigated the feasibility of developing biochar into a termite control agent. Moreover, this approach allowed us to exclude the confounding effects of biochar on the soil environment, thereby evaluating the underlying mechanisms through which biochar ingestion impacts termites.

The *C. formosanus* is a symbiont with a complex symbiotic system in their enlarged hindgut, which helps them to digest lignocellulose [13]. This system comprises a variety of prokaryotes and specialized flagellated protists (protozoa) that are mostly involved in the hydrolysis of cellulose and hemicellulose [14,15]. The host termite and its symbiotic microbiota form an interdependent metabolic unit. Cellulose is fermented by the flagellates to produce acetate, CO_2_, and H_2_, which are then used by host-associated bacteria for nitrogen fixation, acetogenesis, and other processes, ultimately providing nutrition to the termite [16,17]. Any disturbance to such a delicate symbiotic relationship, especially with the flagellate community, may prove catastrophic for the termite, resulting in starvation and death [18]. Since gut symbionts play a crucial role in the physiology of termites, biochar consumption is assumed to exert a toxic effect by interrupting this crucial symbiotic system.

Therefore, in this study, we conducted three sets of experiments: First, we measured the effects of biochar ingestion on termite fitness (body weight and mortality) to assess the potential of developing biochar as a termite control agent. Second, we characterized the concomitant changes in the termite gut microbial community (including both the bacterial community and the symbiotic flagellate community) to determine whether the impact of biochar on termites is associated with disruption of this symbiotic system. Finally, we employed transcriptomic analysis to generate hypotheses about host metabolic and stress-response pathways associated with biochar ingestion, aiming to identify possible mechanistic links rather than to establish a continuous causal cascade.

## 2. Materials and Methods

### 2.1. Biochar and Feed Preparation

Biochar was purchased from Nanjing Qinfeng Straw Technology Co. Ltd., Nanjing, China, where it was produced by the pyrolysis (550–650 °C without oxygen) of rice straw.

To facilitate ingestion by termites, biochar powder was mixed with pine wood powder at dry mass ratios of 0 (control), 5 and 20%, constituting three biochar amendment treatments. 50 µL of ultrapure water were added to each 100 mg of the mixture to make it adhere. Then, it was pressed into feed tablets (diameter 10 mm, height 3 mm) using a press machine.

### 2.2. Termite Collection and Rearing

Three colonies of *C. formosanus* were used in the experiments, and all of them were collected using a lure collection device (homemade, one plastic basket with holes and a pile of pine blocks in it) from the Longdong Forest (Guangzhou, China). Each colony is more than 1 km apart to prevent the collected termites from a same colony. Stock colonies were fed with wet pinewood and maintained in the dark in a greenhouse (26 ± 1 °C and 70–80% relative humidity). All colonies were maintained no more than 1 month before the experiment.

### 2.3. Food Choice Experiment

To test whether termites exhibit a feeding preference for food mixed with biochar, we placed two food blocks on opposite sides of the same Petri dish (10 cm in diameter). The control side contained a block made of pure pine wood powder (0% biochar), while the treatment side contained a block mixed with pine wood powder at concentrations of 0%, 5%, or 20% biochar. Ten worker termites were placed in each Petri dish. Each treatment was replicated across 20 Petri dishes. After 2 days, the degree of feeding on the food blocks was assessed. Based on the extent of damage, feeding was categorized into five levels: 1) The food block remained largely intact with only sporadic, minor feeding; assigned a feeding score of 0.2. 2) Obvious feeding marks appeared, with less than half of the block damaged; assigned a score of 0.4. 3) More than half of the block was consumed or damaged, but a small portion remained intact; assigned a score of 0.6. 4) Only a small portion of the block remained undamaged; assigned a score of 0.8. 5) The entire block was consumed or destroyed; assigned a score of 1.0.

### 2.4. Termite Fitness

The experimental unit consisted of a Petri dish (10 cm in diameter) containing a glass coverslip in the center, on which three identical feed tablets were placed. All three tablets in a given dish contained the same biochar amendment level (0%, 5%, or 20%). Feed tablets were replaced at 5-day intervals.

#### 2.4.1. Mortality Assay

We added 30 workers of similar sizes into each Petri dish, and all Petri dishes were maintained in a dark greenhouse (26 ± 1 °C and 70–80% relative humidity). Termite mortality was assessed on days 10, 20 and 30. Each treatment group was repeated 20 times.

#### 2.4.2. Body Weight Measurement

We added 100 workers of similar sizes into each Petri dish, and all of the Petri dishes were maintained in a dark greenhouse (26 ± 1 °C and 70–80% relative humidity). After 20 days, 50 workers from each Petri dish were weighed at random. Each treatment group was repeated 20 times.

### 2.5. Gut Microbiota Analysis

#### 2.5.1. Bacterial Community

Based on preliminary experiments, we found that the guts of workers in 20 d were too fragile, making it difficult to conduct further measurements of gut bacteria. Therefore, this study selected the 10th day for experimentation. Experiments were conducted with feed tablets containing 0%, 5%, and 20% biochar amendments. The experimental method is the same as that in 2.4. Each treatment group was repeated 4 Petri dishes. Fifty workers at random from each replicate Petri dish were collected from each treatment group. The guts of 50 workers per treatment were dissected using a sterile scalpel. The metagenomic DNA of gut microbes were extracted using TIANamp Genomic DNA Kits (Tiangen, Beijing, China) following the manufacturer’s instructions. The purity of DNA was quantified using a NanoDrop spectrophotometer (Thermo Fisher Scientific, Wilmington, DE, USA), and the quality and integrity of DNA were assessed using 1.0% agarose gel electrophoresis. The bacterial 16S rRNA gene was amplified using the 27F (5′-AGRGTTTGATYNTGGCTCAG-3′) and 1492R (5′-TASGGHTACCTTGTTASGACTT-3′) primers. Purified PCR products were pooled and sequenced on a PacBio Sequel II platform (Biomarker Technologies, Beijing, China). CCS reads were generated using SMRT Link v8.0 (minPasses ≥ 5, minPredictedAccuracy ≥ 0.9), demultiplexed by barcode with lima v1.7.0, trimmed and length-filtered (1200–1650 bp) with cutadapt v1.9.1, and cleaned of chimeras using UCHIME v4.2. Effective-CCS sequences, all with lengths of approximately 1.5 kb. Rarefaction and Shannon index curves confirmed adequate sequencing depth. OTUs were clustered at 97% similarity using UPARSE (v10.0); OTUs representing < 0.005% of total reads were discarded. Taxonomic assignment was performed against the SILVA database (v138) using the QIIME2 naive Bayes classifier (confidence threshold 0.7) [19]. Alpha diversity indices (ACE, Chao1, Shannon, Simpson, PD_whole_tree, Coverage) and beta diversity (Bray–Curtis and Jaccard distances) were calculated using QIIME2. Community dissimilarities were visualized via PCoA, NMDS, and UPGMA clustering. Differences between treatments were assessed using PERMANOVA (Adonis, 999 permutations) and ANOSIM (vegan R package). Taxa with differential abundance were identified using LEfSe (LDA > 4), MetagenomeSeq (v1.22.0), and Metastats (*p* < 0.05).

#### 2.5.2. Flagellate Community

Feed tablets with 0%, 5%, and 20% biochar amendments were used. The experimental method is the same as that in 2.4. A total of 20 termites were randomly selected from the 3 Petri dishes of each treatment for individual replicates. The enlarged hindgut wall of each termite was punctured with a dissecting needle and immediately immersed in a 1.5 mL centrifuge tube containing 100 μL of Solution U, placed on ice. Under a microscope, the numbers of the three flagellate species (*Holomastigotoides mirabile*, *Pseudotrichonympha grassii* and *Spirotrichonympha leidyi*) were counted in a hemocytometer, and the total number of flagellates was calculated based on the total volume of the gut contents [20].

### 2.6. Termite RNA Sequencing and Transcriptomic Analysis

The experimental method is the same as that in 2.4. Experiments were conducted with feed tablets containing 0%, 5%, and 20% biochar amendments. Each treatment group was repeated 3 Petri dishes. Eight workers from the same Petri dish were pooled together in each sample, and total RNA was extracted using TRIzol (Invitrogen, Carlsbad, CA, USA). RNA integrity was assessed using an Agilent 2100 Bioanalyzer (Agilent Technologies, Santa Clara, CA, USA). cDNA libraries were constructed and sequenced on an Illumina NovaSeq 6000 platform (2 × 150 bp paired-end) (Biomarker Technologies, Beijing, China). Approximately 7.89 Gb of clean data was generated per sample, with Q30 bases ≥ 92.61%. Raw reads were filtered to remove adapters and low-quality reads (Q ≤ 10 or containing > 10% N). The clean reads were mapped to the *C. formosanus* reference genome (NCBI assembly GCA_013340265.1, CopFor1.0) using HISAT2. Gene expression levels were quantified as fragments per kilobase of transcript per million mapped reads (FPKM) using StringTie. Differentially expressed genes (DEGs) were identified using DESeq2 with thresholds of fold change ≥ 1.5 and a false discovery rate (FDR) < 0.01. Functional enrichment of DEGs was performed using the KEGG database via clusterProfiler.

### 2.7. Data Analysis

To test whether termites exhibit a feeding preference for food mixed with biochar, the data were divided into two treatments. One was the amount of biochar pine wood powder (0, 5% and 20%), while another was biochar treatment (pine wood powder and biochar + pine wood powder). Each device repeat was independent. And the feeding scores are decimal values between 0 and 1; therefore, a generalized linear model (Binomial distribution and log link function) was used for the analysis. Termite mortality was the binary data. Therefore, a generalized linear model (Binomial distribution and log link function) was used to examine the data. The number of flagellate was a non-negative integer, so a generalized linear model (quasi-Poisson error structure, log link function) was used to examine the data since they were over-dispersed [21]. A one-way analysis of variance (ANOVA) was used to examine the termites’ body weight. Residual plots were used to visual inspection of homoscedasticity and normality and did not reveal any obvious deviations. After detecting a significant effect, the Tukey post hoc comparison procedure was used to make pairwise contrasts between treatments using the emmeans R package [22]. All data analyses were run with R 4.0.2 statistical software [23], and figures were created by the R package ggplot2 [24].

## 3. Results

### 3.1. Feeding Score in Each Treatment in Food Choice Experiment

**Figure 1:**
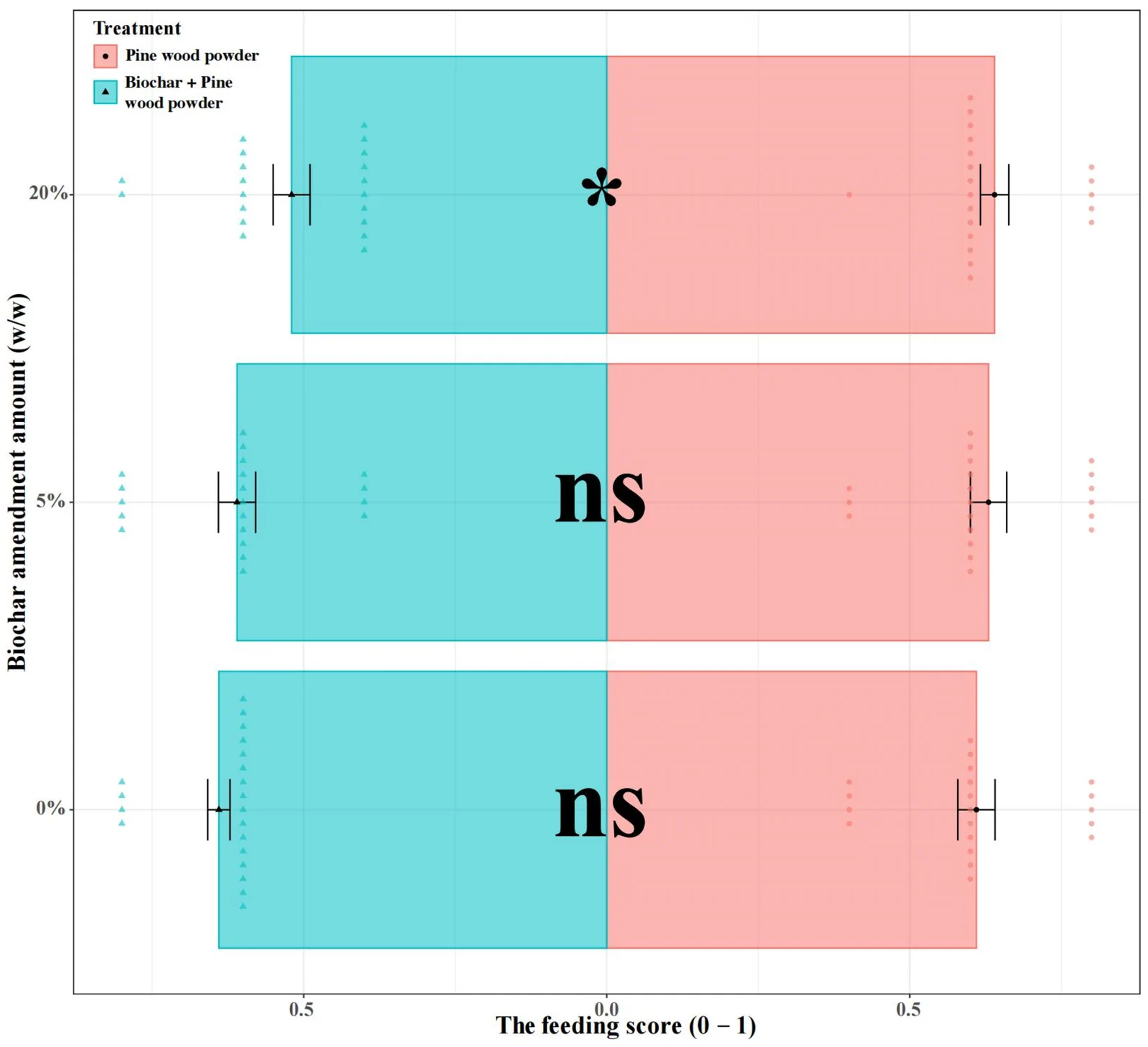
Mean (±SE) feeding score in each treatment in food choice experiment. Note: The asterisk indicates a significant difference and ns indicates no significant difference between left and right compared treatment at α = 5%.

The degree of feeding on the food blocks in each treatment was not significantly affected by the amount of biochar (χ^2^ = 0.79, *p* = 0.37) and biochar treatment (χ^2^ = 3.69, *p* = 0.16), but was significantly affected by their interaction (χ^2^ = 7.43, *p* = 0.02) (Figure 1). In the food with 0% and 5% amounts of biochar, there were no significant differences between selective pure pine wood powder or biochar + pine wood powder (z = 0.77, *p* = 0.44; z = 0.51, *p* = 0.61, respectively). And in 20% treatment, the feeding score in 20% was significantly lower than pure pine wood powder treatment by 18.75% (z = 3.02, *p* = 0.003).

### 3.2. Biochar Exposure Reduces Termite Body Weight and Survival

**Figure 2:**
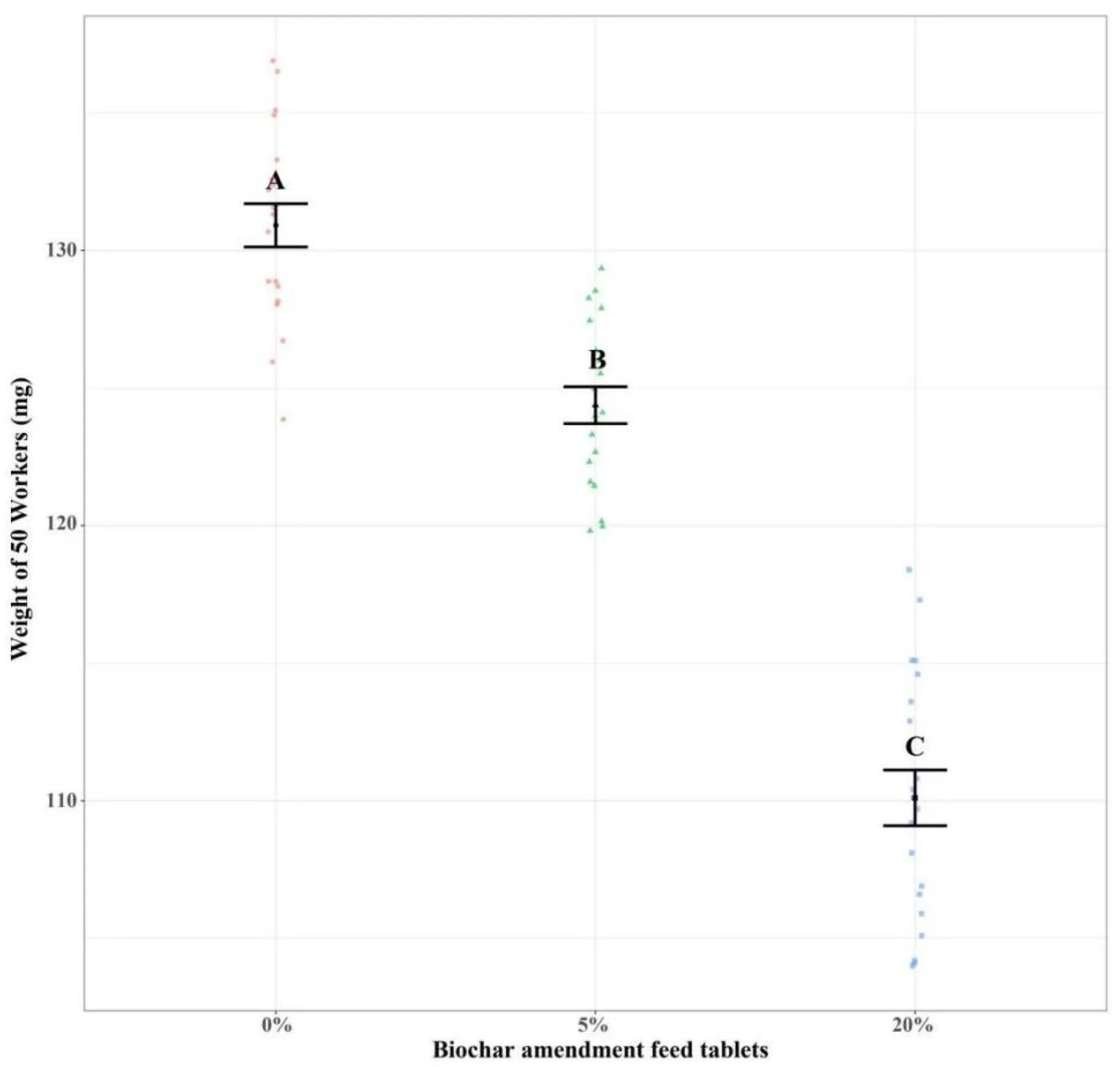
Mean (±SE) weight (mg) of 50 workers. Note: Different capital letters indicate significant differences between biochar amendment amounts according to Tukey’s HSD test (*p* < 0.05). The same below.

Termites’ body weight was significantly affected by the amount of biochar added (F_2,57_ = 162.29, *p* < 0.001) (Figure 2). The weight of termites in the 20% treatments decreased significantly by 20.80 mg (t = −17.62, *p* < 0.001) and 6.53 mg (t = −12.08, *p* < 0.001) compared with 0% and 5%, respectively. The weight of termites in the 5% treatments decreased significantly by 6.53 mg (t = −5.54, *p* < 0.001) compared with the 0%.

**Figure 3:**
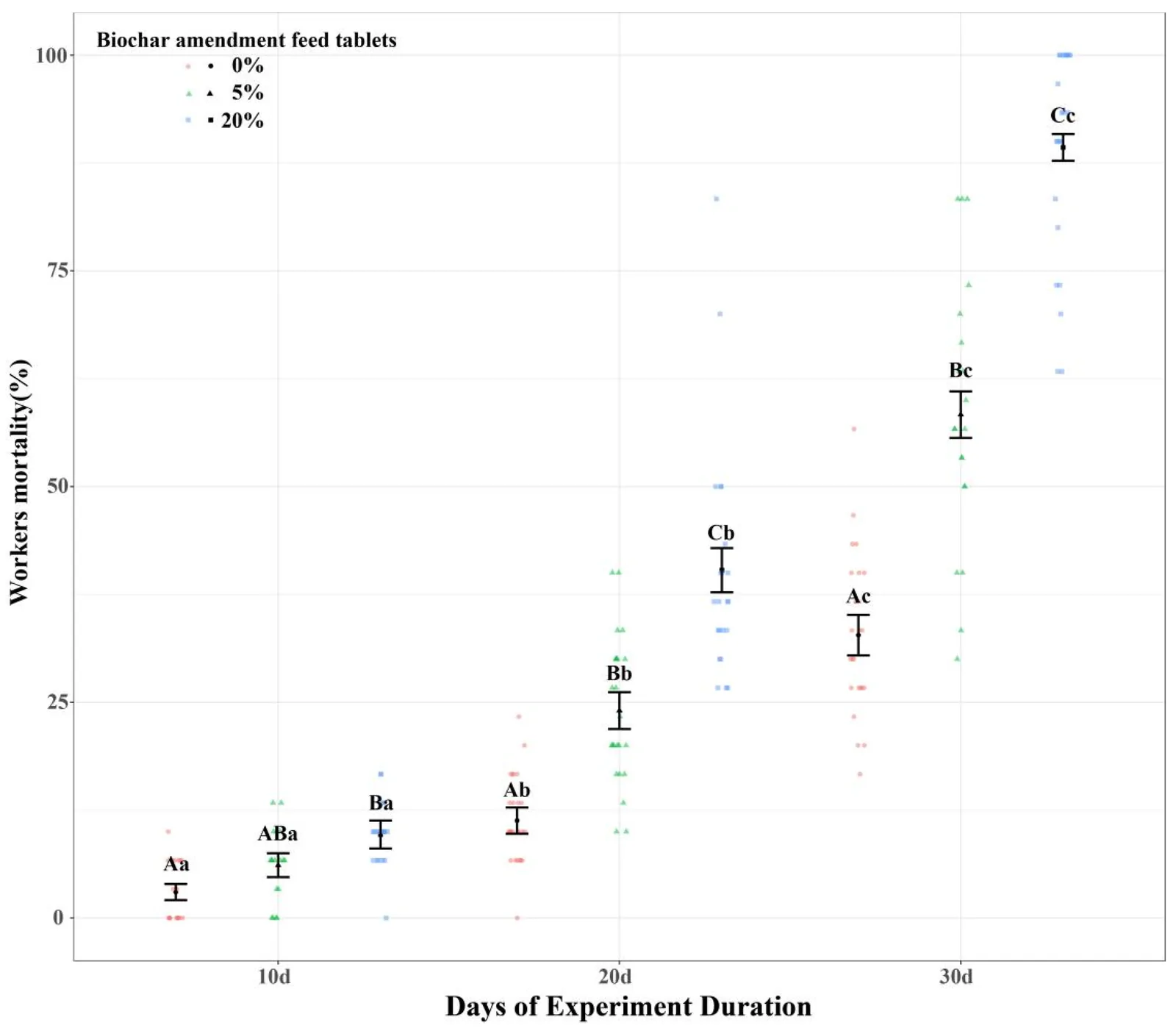
Mean (±SE) mortality of termites. Note: Different capital letters indicate significant differences between biochar amendment amounts in the same duration according to Tukey’s HSD test (*p* < 0.05), and different lowercase capital letters indicate significant differences between duration in the same biochar amendment.

The mortality of termites was significantly affected by the amount of biochar added (χ^2^ = 366.83, *p* < 0.001), the experimental duration (χ^2^ = 880.16, *p* < 0.001), and their interaction (χ^2^ = 31.93, *p* < 0.001) (Figure 3). In the 10 d, the mortality of termites in the 20% treatment increased significantly by 2.22-fold (z = 3.35, *p* = 0.002) compared with 0%. However, compared with the 5% treatment, the mortality of termites in both the 0% and 20% treatments showed no significant difference (z = 1.86, *p* = 0.15; z = 1.64, *p* = 0.23, respectively). In the 20 d, the mortality of termites in the 20% treatment increased significantly by 2.57-fold (z = 9.01, *p* < 0.001) and 0.68-fold (z = 4.80, *p* < 0.001) compared with 0% and 5%, respectively. And the mortality in the 5% increased significantly by 1.13-fold (z = 4.76, *p* < 0.001) compared with 0%. In the 30 d, the mortality of termites in the 20% treatment increased significantly by 1.46-fold (z = 14.68, *p* < 0.001) and 0.53-fold (z = 9.11, *p* < 0.001) compared with 0% and 5%, respectively. And the mortality in the 5% increased significantly by 0.78-fold (z = 6.86, *p* < 0.001) compared with 0%.

### 3.3. Gut Bacterial Community Structure in Termites

**Figure 4:**
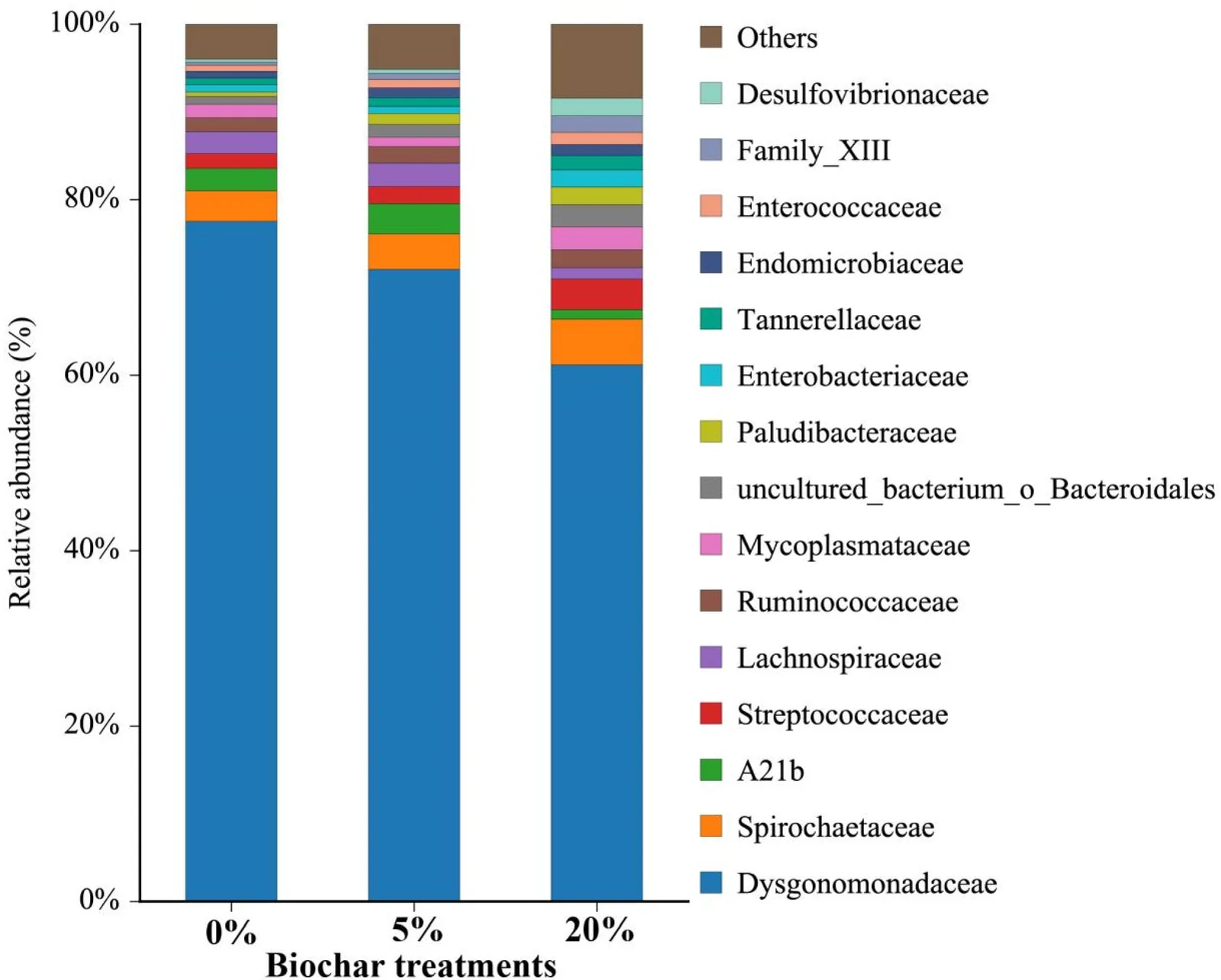
Relative abundance (%) of the top 15 bacterial families in the *C. formosanus* gut under different treatments.

At the family level, the composition also changed notably (Figure 4). The family Dysgonomonadaceae (Bacteroidota), which constituted the dominant core microbiota (77.5% in 0% treatment), exhibited a significant and consistent decrease in relative abundance with increasing treatment dosage (72.1% in Low, 61.2% in 20% treatment). Concurrently, several bacterial families showed a marked increase in the 20% treatment group compared to 0 and 5% treatment groups. The most notable increases were observed in: Spirochaetaceae (5.21% in 20% treatment vs. 3.49% in 0% treatment), Streptococcaceae (3.48% in 20% treatment vs. 1.68% in 0% treatment), Enterobacteriaceae (1.95% in 20% treatment vs. 0.82% in 0% treatment), Desulfovibrionaceae (2.03% in 20% treatment vs. 0.34% in 0% treatment), Mycoplasmataceae (2.61% in 20% treatment vs. 1.51% in 0% treatment) and Tannerellaceae (1.62% in 20% treatment vs. 0.75% in 0% treatment). The ‘Others’ category also increased substantially in the 20% treatment (8.42% vs. 3.98% in 0% treatment).

### 3.4. Biochar Causes a Severe Depletion of Symbiotic Flagellates

**Figure 5:**
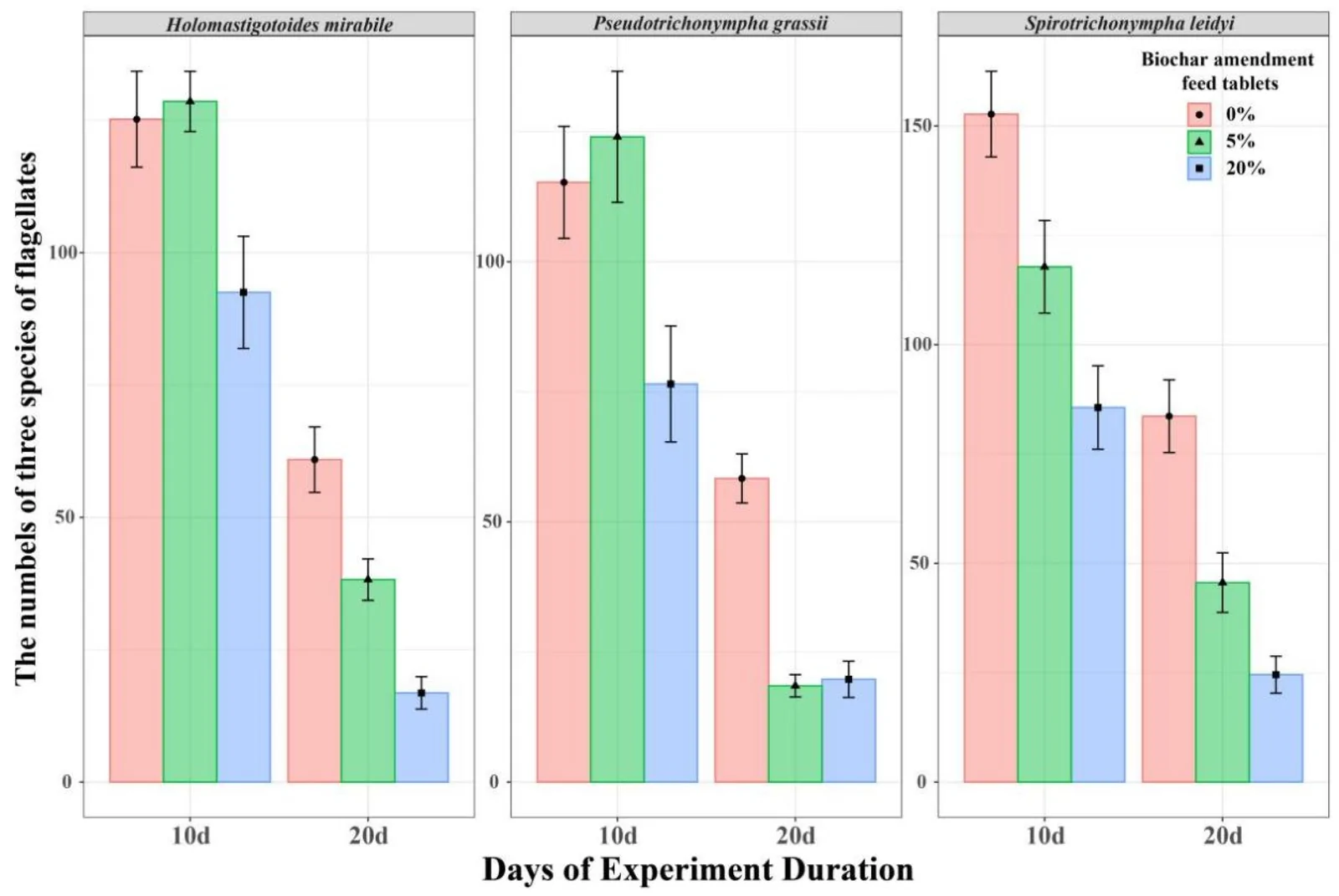
Mean (±SE) numbers of three symbiotic flagellates.

The numbers of *H. mirabile* were significantly affected by the amount of biochar added (χ^2^ = 32.75, *p* < 0.001), the experimental duration (χ^2^ = 180.59, *p* < 0.001) and their interaction (χ^2^ = 17.74, *p* < 0.001) (Figure 5). In the 10 d, the numbers of *H. mirabile* in the 20% treatment decreased significantly by 32.70 (z = −2.81, *p* = 0.01) and 36.05 individuals (z = −3.01, *p* = 0.006) compared with 0% and 5%, respectively. There was no significant difference between 5% and 0% (z = 0.25, *p* = 0.97). In the 20 d, the numbers of *H. mirabile* in the 20% treatment decreased significantly by 44.05 (z = −5.64, *p* < 0.001) and 21.40 individuals (z = −3.39, *p* = 0.002) compared with 0% and 5%, respectively. The numbers of *H. mirabile* in the 5% treatment decreased significantly by 22.65 individuals (z = −2.73, *p* = 0.02) compared with 0%.

The numbers of *P. grassii* were significantly affected by the amount of biochar added (χ^2^ = 25.74, *p* < 0.001), the experimental duration (χ^2^ = 132.16, *p* < 0.001) and their interaction (χ^2^ = 20.67, *p* < 0.001) (Figure 5). In the 10 d, the numbers of *P. grassii* in the 20% treatment decreased significantly by 38.75 (z = −3.01, *p* = 0.007) and 47.50 individuals (z = −3.60, *p* < 0.001) compared with 0% and 5%, respectively. There was no significant difference between 5% and 0% (z = 0.58, *p* = 0.83). In the 20 d, the numbers of *P. grassii* in the 5% and 20% treatment decreased significantly by 39.85 (z = −4.23, *p* < 0.001) and 38.60 individuals (z = −4.28, *p* < 0.001) compared with 0%, respectively. There was no significant difference between 5% and 20% (z = 0.21, *p* = 0.98).

The numbers of *S. leidyi* were significantly affected by the amount of biochar added (χ^2^ = 53.65, *p* < 0.001), the experimental duration (χ^2^ = 92.70, *p* < 0.001) and their interaction (χ^2^ = 7.62, *p* = 0.02) (Figure 5). In the 10 d, the numbers of *S. leidyi* in the 20% treatment decreased significantly by 67.07 (z = −4.74, *p* < 0.001) and 32.17 individuals (z = −2.47, *p* = 0.04) compared with 0% and 5%, respectively. There was no significant difference between 5% and 0% (z = 2.21, *p* = 0.07). In the 20 d, the numbers of *S. leidyi* in the 20% treatment decreased significantly by 59.10 (z = −5.58, *p* < 0.001) and 21.05 individuals (z = −2.58, *p* = 0.03) compared with 0% and 5%, respectively. The numbers of *S. leidyi* in the 5% treatment decreased significantly by 38.05 individuals (z = −3.44, *p* = 0.002) compared with 0%.

### 3.5. Transcriptomic Responses Reveal Suppressed Metabolism and Activated Stress Pathways

Because several enriched pathways did not remain significant after multiple-testing correction, the KEGG enrichment results are interpreted below as exploratory trends rather than confirmatory mechanistic evidence.

**Figure 6:**
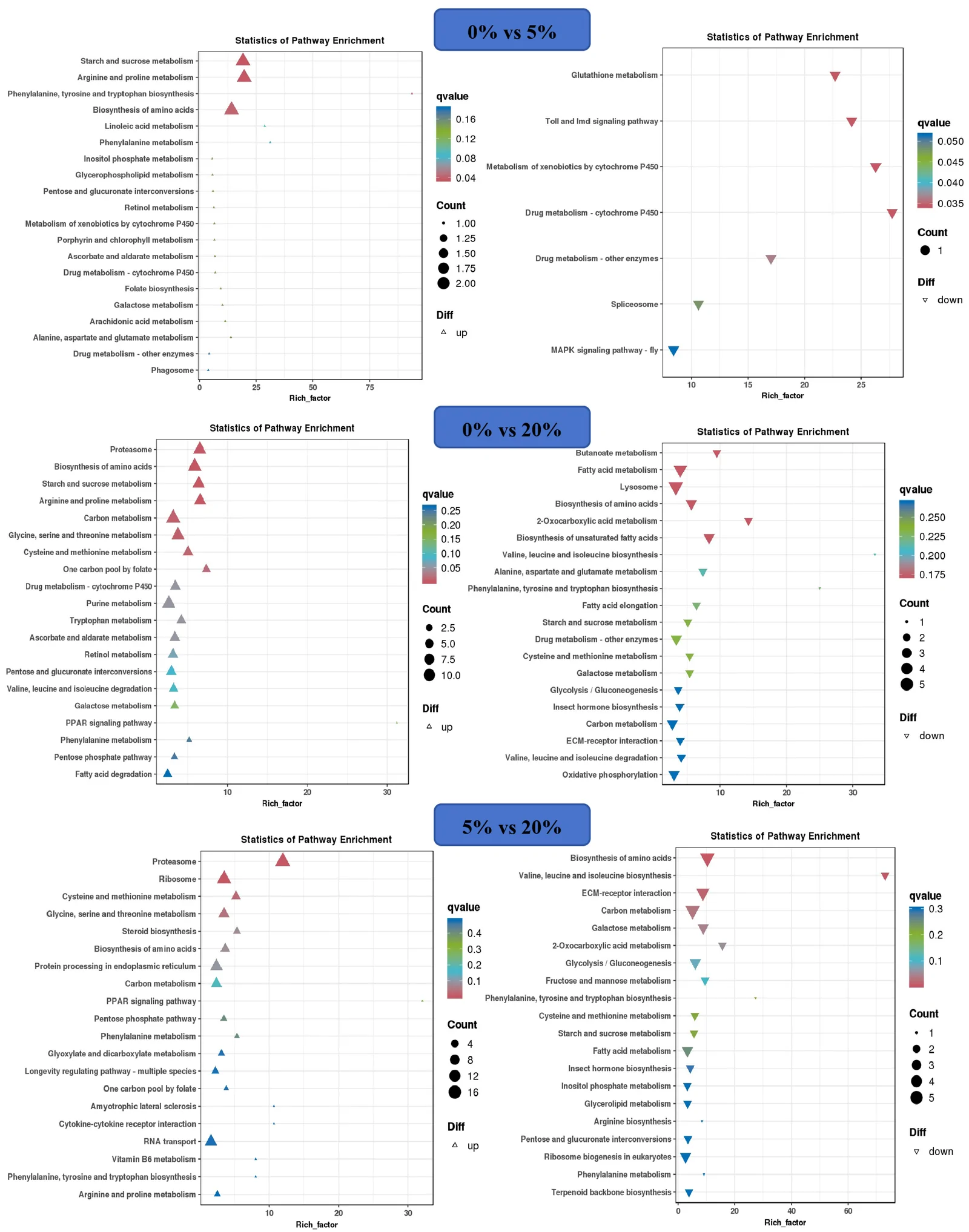
The top 25 KEGG pathways enriched for differentially expressed genes among different treatments.

The number of differentially expressed genes among treatments is shown in Table 1. The top 25 KEGG pathways enriched for differentially expressed genes among different treatments are shown in Figure 6.

0% vs. 5%: This low-dose contrast produced only a small DEG set (45 total; 40 up, 5 down). Enriched up-terms were scattered across xenobiotic-detoxification categories (cytochrome P450-related metabolism, glutathione/other drug-metabolizing terms), selected amino-acid pathways (e.g., arginine/proline, general amino-acid biosynthesis), and carbohydrate/energy terms (starch/sucrose, galactose). A minor set of immune/xenobiotic terms, including Toll/Imd-related and certain P450 groupings, appeared among down-regulated entries. Overall enrichment was weak, with low gene counts per pathway, indicating that 5% biochar elicited only a diffuse transcriptional alert rather than coordinated reprogramming.

0% vs. 20%: This high-dose contrast generated the largest DEG set (618 total; 424 up, 194 down). Up-regulated pathways concentrated on protein turnover/degradation, amino-acid catabolism, cellular stress/Detoxification, and partial carbon/nitrogen salvage (amino-acid biosynthesis, starch/sucrose, purine). Down-regulated pathways concentrated on translation and RNA processing (ribosome, RNA transport, ribosome biogenesis), protein-folding/quality control (endoplasmic-reticulum processing), and central energy metabolism (overall carbon metabolism, glycolysis/gluconeogenesis, fatty-acid metabolism, biosynthetic amino-acid routes). The reciprocal pattern indicates a systemic shift from growth-associated anabolism to stress-associated catabolism.

5% vs. 20%: This contrast yielded 491 DEGs (318 up, 173 down) and an enrichment structure broadly similar to 0% vs. 20%. Up-terms again covered carbon/amino-acid catabolism, detoxification, purine metabolism, and additional proteostatic/recycling terms including autophagy; down-terms again covered ribosome, RNA transport, ER processing, carbon metabolism, and amino-acid biosynthesis. The similarity between 0% vs. 20% and 5% vs. 20% indicates that the catabolic/anabolic imbalance is already initiated at a lower dose and amplified at 20%.

In summary, biochar ingestion induced a dose-dependent transcriptomic continuum: negligible/diffuse stress signals at 5%, and coordinated up-regulation of degradation/detoxification/catabolism with concurrent down-regulation of translation and central energy metabolism at 20%.

## 4. Discussion

### 4.1. Bait Potential and Feeding Behaviour

The food-choice data show that 5% rice-straw biochar in pine-wood powder did not significantly reduce intake, whereas 20% lowered the feeding score by ~18.75%. For subterranean-termite baits, an ideal active should be ingested reliably, act slowly, and be transferred among nestmates by trophallaxis before widespread mortality; chitin-synthesis-inhibitor baits such as noviflumuron achieve colony elimination even after short feeding because the toxicant is shared and lethal time is not strictly dose-proportional [25]. The mortality trajectory in this study—progressive from day 10 to 30, more consistent with metabolic inhibition than acute neurotoxicity—fits a bait-oriented rather than contact-insecticide profile. Notably, 5% biochar already caused significant mortality by day 30 despite a small transcriptomic response, indicating that gut-symbiotic disruption can precede whole-body transcriptional reorganization. The 20% reduction in intake is not prohibitive but is large enough to delay bait uptake; particle-size reduction, microencapsulation, phagostimulants, or lowering the active load with adjuvants would improve palatability [9,10]. However, we did not quantify actual food consumption in the no-choice exposure experiment, and no pair-fed or inert-particle control was included. Therefore, reduced substrate intake and dietary dilution remain explicit alternative explanations, especially for the 20% treatment. The 5% diet was non-deterrent and still caused mortality, suggesting that factors beyond palatability contribute, but this does not exclude intake effects. Direct physicochemical toxicity should therefore be regarded as a hypothesis rather than the established primary mechanism.

**Figure A1:**
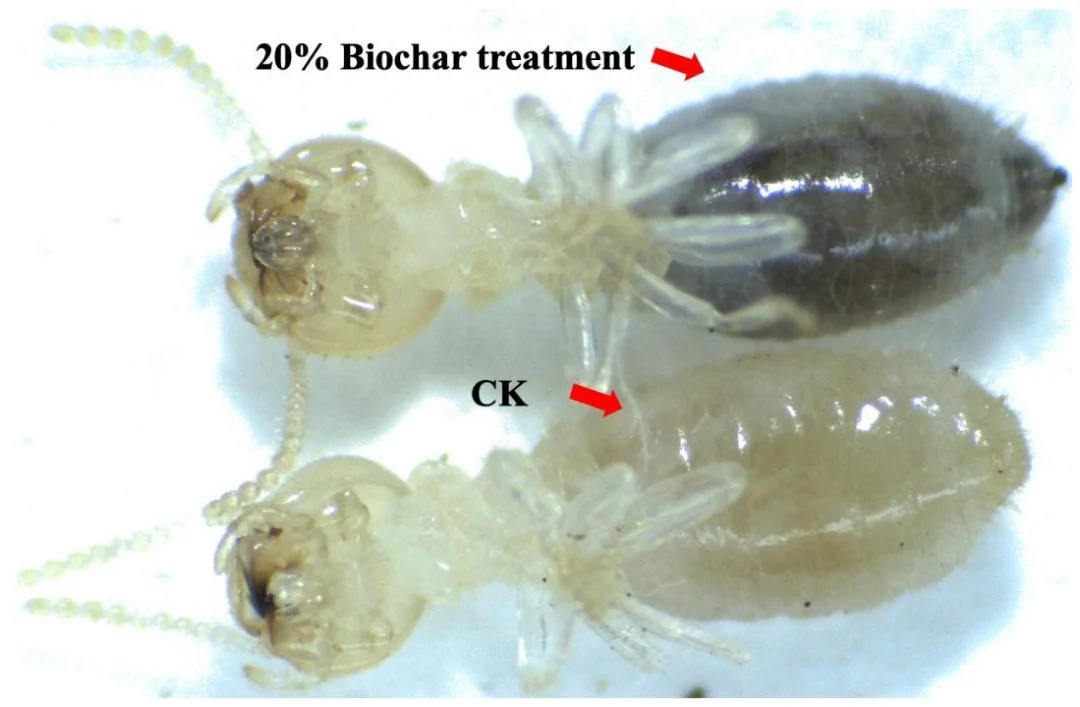
Abdominal morphology of termites fed different biochar tablets. Note: upper termite fed the 20% biochar-amended tablets, the red arrow points to its blackened abdomen; lower termite fed the control tablets without biochar, the red arrow points to the abdomen showing no color change.

**Figure A2:**
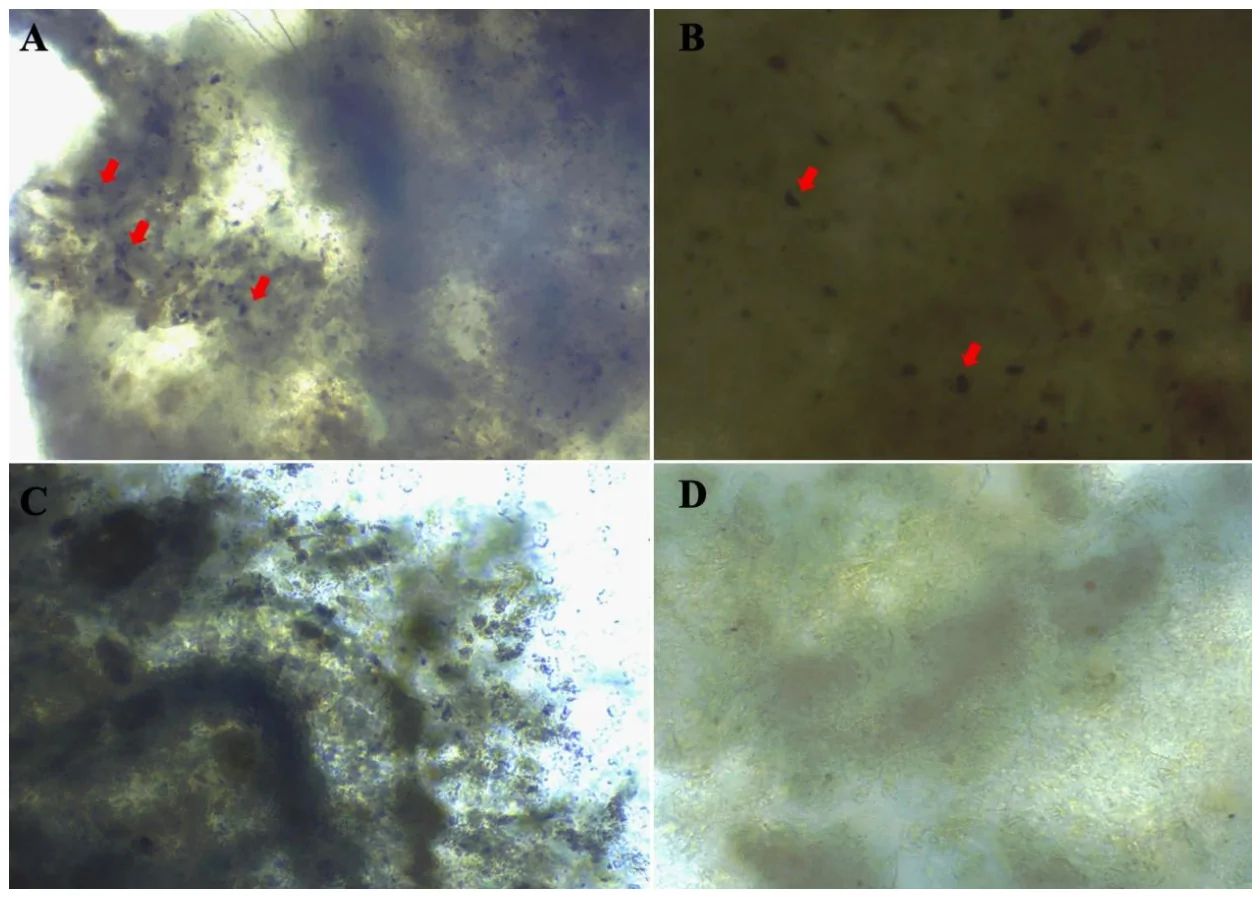
Gut contents of termites fed different diets. Note: (**A**,**B**) Termites fed the 20% biochar-amended diet. (**B**) is an enlarged view of the area in (**A**), with red arrows indicating fine biochar particles. (**C**,**D**) Termites fed the control diet without biochar. (**D**) is an enlarged view of the area in (**C**), showing no black particles.

The observed substantial reduction in body weight and survival of termites fed biochar-amended diets is consistent with comparable results in other soil invertebrates, such as earthworms and collembolans [9,26]. We discovered a significant amount of fine biochar particles within the gut of termites (Figure A1 and Figure A2). Fine biochar particles may pose two types of hazards: first, some studies suggest that these particles can damage the gut epithelium [10,26]; secondly, and more critically, based on extensive literature characterizing biochar produced from similar feedstocks and temperatures [27], toxic substances such as polycyclic aromatic hydrocarbons (PAHs), persistent free radicals (PFRs), and heavy metals are known to be generated and concentrated during the pyrolysis process. Upon direct ingestion by termites, these chemical toxicants first come into contact with and act on the intestinal tissues and the resident symbiotic microbes. However, we did not directly measure these specific toxicants in our biochar batch. Their involvement therefore remains a plausible, unmeasured hypothesis grounded in established biochar chemistry, not a demonstrated component of the proposed mechanism. Likewise, adsorption effects and changes in gut physicochemistry were not measured and require future targeted investigation.

We acknowledge that replacing 20% of the dietary wood powder with biochar dilutes the nutritional content of the feed and could theoretically affect palatability. However, several lines of evidence argue against reduced feeding being the sole or primary driver of the observed effects. First, our previous soil-based study [11] demonstrated that *C. formosanus* fitness was significantly reduced in soil containing >10% biochar, even though termites in soil have access to abundant untreated wood sources, indicating that the effect is not merely a consequence of a diluted artificial diet. Second, the specific and profound collapse of the flagellate community (Figure 6) and the activation of detoxification pathways (Figure 5) are hallmarks of a direct toxic insult rather than simple caloric restriction. Starvation typically leads to a general suppression of metabolic activity, not the specific upregulation of cytochrome P450 and glutathione metabolism pathways observed here. Nevertheless, because intake was not quantified and no pair-fed or inert-particle control was included, we cannot exclude intake reduction as a contributing or alternative explanation. The current data therefore support a working hypothesis of direct physicochemical toxicity but do not establish it as the primary mechanism. Future studies should quantify consumption, include pair-fed and inert-particle controls, and test washed biochar/leachate and specific toxicants.

### 4.2. The Dramatic Decline of Flagellate Protist Communities

In lower termites, such as *C. formosanus*, symbiotic flagellated protists living in the hindgut are the major microbes responsible for degrading lignocellulose [14]. The findings of this study revealed that high-dose biochar treatment resulted in a sharp and significant decrease in populations of three major cellulolytic flagellates (*H. mirabile*, *P. grassii*, and *S. leidyi*) (Figure 5). Because the flagellate, bacterial, and host transcriptomic responses were measured in parallel rather than in a continuous causal experiment, this decline should be described as a key associated event in the proposed cascade, not as an established first causal step.

The decline in flagellates can be explained by two non-exclusive processes. First, the biochar particles, or the chemical toxins leached from them, can have direct toxicity on the membranes of flagellates, organelles, or metabolic enzymes, causing their death or lysis [10]. Second, the adsorptive capacity of biochar could modify the physicochemical conditions of the gut material (e.g., pH, redox potential) or adsorb some trace nutrients vital to the growth of flagellates, providing an unfavorable microenvironment to the flagellates [10]. However, this study failed to distinguish the relative roles of these two mechanisms, which should be explored in future studies. Regardless of the cause, the collapse of the flagellate community would be expected to directly lead to a precipitous drop in the capacity to ferment lignocellulose, thereby presumably severing the termite’s most important energy source [14]. This provides a plausible explanation for the observed trend toward downregulation of host transcriptomic pathways related to energy acquisition and utilization, such as butanoate metabolism and fatty acid metabolism, as these host pathways would be expected to be downregulated in response to a reduced supply of microbial fermentation products like acetate. However, since these enrichment results did not reach statistical significance after correction, this interpretation remains speculative and requires further validation. We therefore treat the flagellate decline as a key associated event and a working hypothesis, rather than as confirmed causal evidence.

### 4.3. Perturbation of Core Bacterial Symbionts Is Associated with Potential Nutritional and Energy Stress

**Figure A3:**
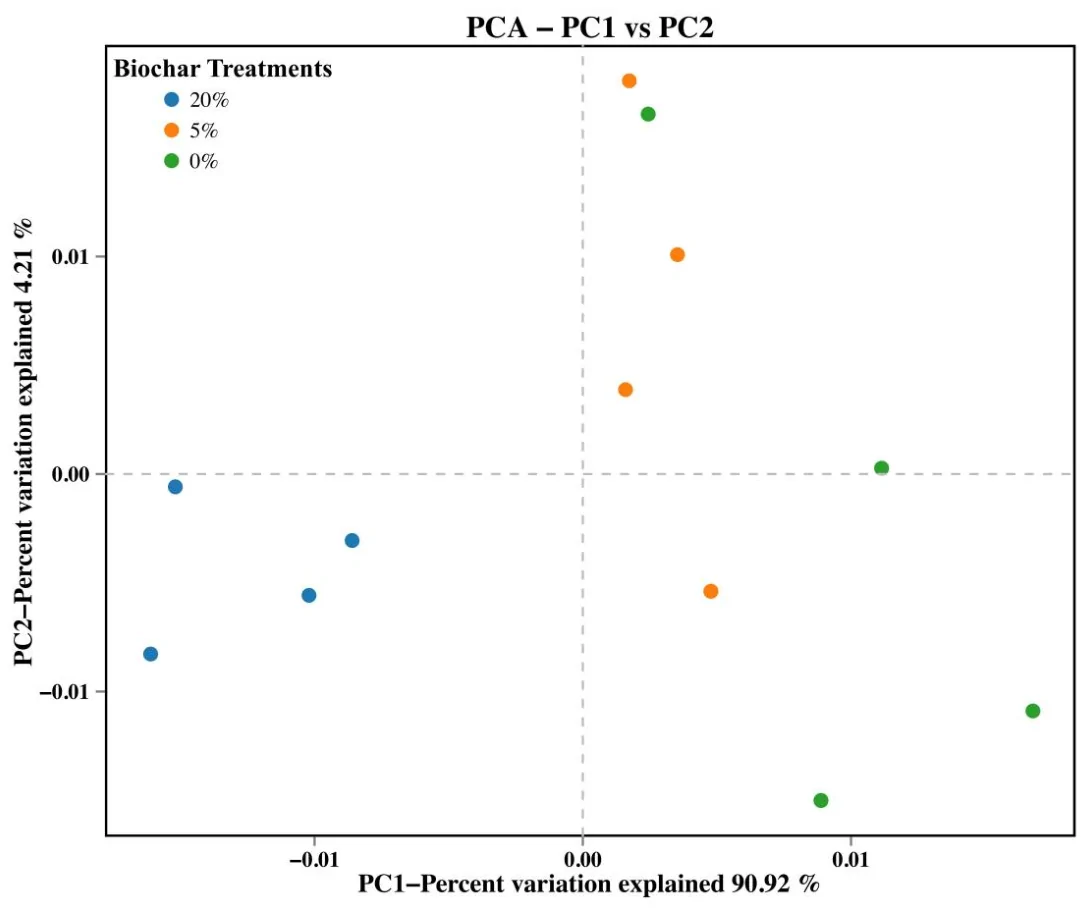
Principal components analysis of the gut bacterial community of *C. formosanus.*

**Figure A4:**
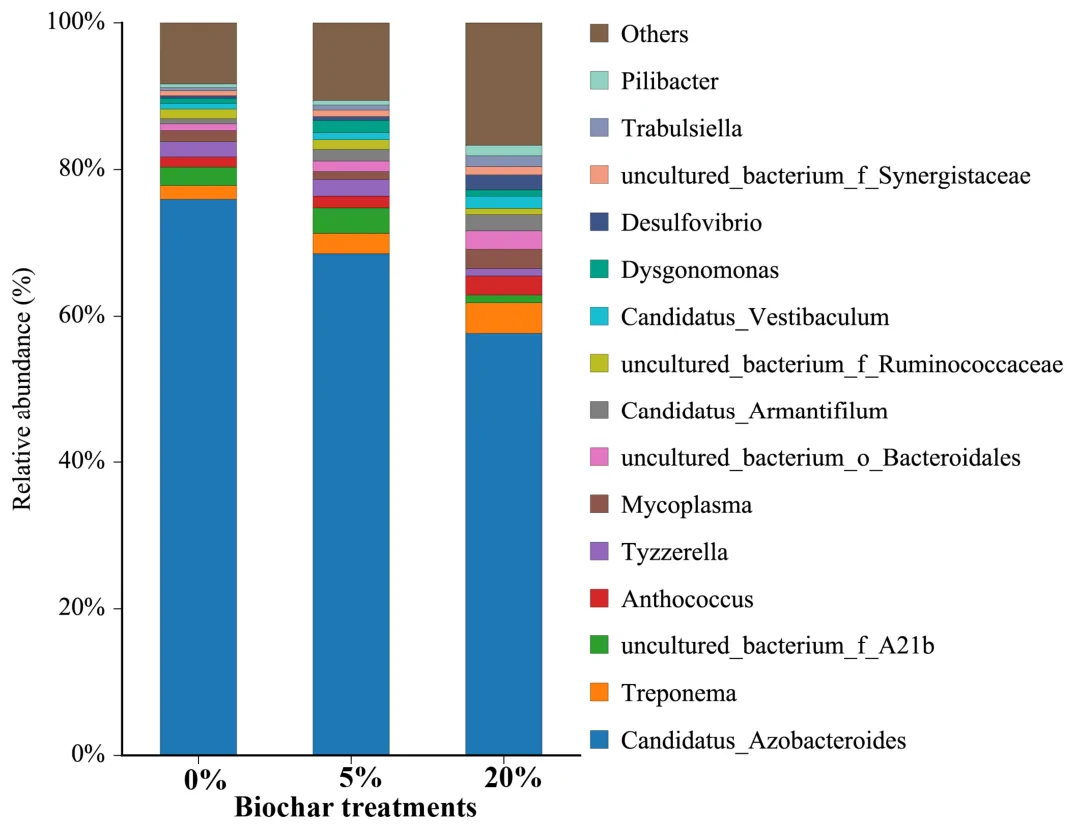
Relative abundance (%) of the top 15 bacterial genera in the termite gut under different treatments.

The gut bacterial community of *C. formosanus* was significantly reshaped by biochar ingestion and showed clear separation among the 0, 5, and 20% treatment groups at the family level (Figure A3). The observed shifts in the gut bacterial community structure in this study are not random fluctuations but a reorganization coupled with flagellate decline and possessing clear metabolic functional implications (Figure 4 and Figure 5). The synchronous and significant decrease in the abundance of *Candidatus* Azobacteroides (belonging to the family Dysgonomonadaceae) (Figure A4), an endosymbiont of flagellates, is consistent with the reduction in its host protist population [13]. This bacterium is a known efficient diazotroph, capable of converting atmospheric nitrogen into ammonia, providing a usable nitrogen source for both the flagellates and the termite host [16]. The decline in its relative abundance is consistent with the hypothesis of a severe impairment of this critical symbiotic nitrogen supplementation pathway, which could potentially lead to a state of ‘nitrogen hunger’. This is consistent with the drastic reprogramming of various amino acid metabolic pathways (e.g., glycine, serine, and threonine metabolism) in the host transcriptome, which may indicate the host’s forced adjustment of its endogenous amino acid metabolic network in response to the cutoff of external nitrogen and amino acid input [28]. However, 16S relative-abundance data alone cannot demonstrate reduced nitrogen fixation or nitrogen hunger; these interpretations remain functional hypotheses that require direct measurements such as nitrogenase activity, N incorporation, or metabolite profiling.

At the same time, the enrichment of other multiple groups of bacteria may represent potential compensatory efforts or niche substitutions in the collapsed gut microbiota. The increase in Enterobacteriaceae may represent a potential compensatory response because this family contains numerous known facultative anaerobic diazotrophs [29]. This increase could potentially compensate for the loss of nitrogen fixation capacity. The increase in Desulfovibrionaceae may be related to sulfate reduction, which may indicate a change in sulfur metabolism or redox state in the gut [30]. Spirochaetaceae may also be important because they can potentially utilize H_2_ and CO_2_ (by-products of flagellate fermentation) to form acetate through acetogenesis [31]. With the possible backdrop of declining hydrogen production as a result of flagellate population collapse, the increased relative enrichment of spirochetes may represent a potential adaptation to better recycle the available hydrogen to maintain the acetate supply, the fundamental energy source. However, whether these changes are compensatory remains untested, and they appear unlikely to fully replace core symbiotic functions. This observation aligns with recent work by Zhang et al., which demonstrated that environmentally driven changes in insect-associated microbiota can profoundly alter host responses to external stressors, highlighting the critical role of the microbiome in mediating host resilience [32].

### 4.4. Gut Microbiota Restructuring and Opportunistic-Associated Taxa: A Hypothesized Secondary Health Risk

The change in bacterial community structure from a highly specialized stable state (focused on Dysgonomonadaceae) to a flattened structure with higher proportions of various groups is consistent with substantial community restructuring [29]. In addition to the above-mentioned changes in functional groups, substantial increases were also noted in those groups that are commonly found in low abundance, including Streptococcaceae and Mycoplasmataceae. These taxa may represent opportunistic bacteria normally kept in check by a healthy gut environment, and their increased relative abundance may be a hypothesized indicator of a disturbed gut habitat [29]. Streptococcaceae are mainly fermenters, and their enrichment may indicate a change in the carbon pathway in favor of simpler carbohydrate fermentation. Moreover, the significant increase in the percentage of “others” may indicate the appearance of a significant number of rare or non-resident species, which could make the community even more unstable [33]. This community restructuring may be associated with impaired compensation for the loss of core functions and may impose additional metabolic or immunological strain on termite health through resource competition, destructive metabolites, or host immune responses. However, pathogen load, infection, immune activation, and gut barrier integrity were not measured; therefore, these potential secondary health effects remain hypotheses and do not establish pathogen proliferation or secondary infection.

### 4.5. Host Transcriptional Reprogramming Suggests a Potential Shift from Growth to Stress/Survival Metabolism

The profile of host transcriptomic response provides exploratory evidence that is consistent with the microbial disruption mentioned above and suggests a potential host strategy of prioritizing survival. According to KEGG pathway enrichment data (Figure 6), host gene expression showed exploratory trends that may indicate directional reprogramming. Because several enrichment signals did not remain significant after multiple-testing correction, these pathways are interpreted as hypothesis-generating rather than confirmatory mechanistic evidence.

The pathways associated directly with survival, maintenance, and damage control showed nominal enrichment. Nominal enrichment was observed in the proteasome pathway, which may suggest an increase in the intracellular ubiquitin-proteasome system. In amino acid scarcity, speeding up the breakdown of existing cellular proteins, especially misfolded or non-essential ones, to recycle free amino acids to synthesize proteins needed in life processes or to provide gluconeogenesis fuel, is an extreme yet conserved cellular self-rescue mechanism [34]. At the same time, the increased drug metabolism and cytochrome P450 and glutathione metabolism pathways may indicate that the host cells are putting significant effort into overcoming the xenobiotic chemical toxins introduced by biochar, a prolonged process of detoxification which, in itself, consumes significant energy and reducing power [35]. In Formosan termites, cytochrome P450 and GST genes are inducible by phenobarbital and septic/xenobiotic challenge [36], so the nominal P450/glutathione enrichment is consistent with, but does not prove, chemical biochar constituents as a stress source. These interpretations remain exploratory because several enrichment signals did not pass multiple-testing correction.

The biosynthesis, growth, and long-term investment pathways showed nominal downregulation. The downregulation of Oxidative phosphorylation, a fundamental energy-producing pathway, may represent a potential response of energy throttling by the host in response to insufficient energy supply, which may be related to the observed weight loss phenotype. Ribosome biogenesis and RNA transport pathway downregulation may represent the scaling back of protein synthesis. The arrest in growth and development may be attributed to the downregulation of the insect hormone biosynthesis pathway from an endocrine view. Moreover, the downregulation of major signaling hubs controlling cell proliferation, differentiation, stress response, and metabolic homeostasis, including the Wnt signaling pathway and FoxO signaling pathway, may indicate that the host paused growth and potentially impaired its stress response and maintenance of homeostasis, redirecting resources towards short-term survival. However, these pathways are exploratory trends and require functional validation.

A useful bait property is protracted action at low dose. Here 5% caused significant weight loss and 30-day mortality with only minor DEGs, whereas protist loss at day 20 and bacterial shifts at day 10 were already evident. This dissociation may suggest a threshold hypothesis: biochar first destabilises flagellate-bacterial symbiosis; host whole-transcriptome reprogramming occurs only after acetate/nitrogen shortage accumulates beyond a repair capacity. However, this threshold hypothesis was not tested by continuous causal experiments. Such microbial-led thresholds, if confirmed, would be preferable in bait development because they decouple “small genomic signal” from “eventual colony-level mortality”, provided uptake is non-deterrent.

Although these lines of evidence are neither continuous nor direct, these exploratory trends are consistent with a hypothesis that: in the dual crisis of energy and nutrition due to the collapse of the symbiotic system, the termite host may shift from growth mode to a survival mode at the expense of the future potential to retain the basic life activities. Such a metabolic transition, if confirmed, would be a multistressor-coordinated response that is intrinsically unsustainable and exhaustive, potentially resulting in physiological dysfunction and death. However, this remains a hypothesis and does not establish a definitive metabolic switch.

## 5. Conclusions

Biochar is a candidate slow-action bait active against *C. formosanus*: 5% was non-deterrent yet caused significant 30-day mortality, while 20% accelerated killing without complete feeding refusal. Our data are consistent with a proposed cascade hypothesis involving initial flagellate decline, subsequent bacterial community restructuring (loss of nitrogen-fixing Dysgonomonadaceae, increased opportunistic-associated taxa), and exploratory host transcriptomic trends from growth toward stress/catabolism (proteasome upregulation, ribosome/energy suppression). However, these links are inferred from parallel measurements and require continuous causal and rescue experiments for confirmation. Reduced intake at 20%, dietary dilution, and unmeasured biochar physicochemical properties remain alternative or non-exclusive explanations. This potential microbial-led threshold, if confirmed, could allow lethal action with minimal early transcriptomic response, a desirable bait property. Future work should quantify consumption, include pair-fed and inert-particle controls, characterize biochar batch chemistry, improve palatability via formulation, conduct nest-level field trials at 5%, and assess non-target safety.

## Figures and Tables

**Table 1:** The number of differentially expressed genes among treatments.

Group	DEGs-Up	DEGs-Down	DEGs-Total
0 vs. 20%	424	194	618
0% vs. 5%	40	5	45
5% vs. 20%	318	173	491

## Data Availability

The original contributions presented in this study are included in the Appendix A. And the 16S OTU representative sequences and differentially expressed gene sequences have been deposited in Zenodo under DOI: 10.5281/zenodo.23088044.
